# Acute Kidney Injury Incidence, Recovery, and Long-term Kidney Outcomes Among Hospitalized Patients With COVID-19 and Influenza

**DOI:** 10.1016/j.ekir.2021.07.008

**Published:** 2021-07-15

**Authors:** Ian A. Strohbehn, Sophia Zhao, Harish Seethapathy, Meghan Lee, Nifasha Rusibamayila, Andrew S. Allegretti, Xavier Vela Parada, Meghan E. Sise

**Affiliations:** 1 Department of Medicine, Division of Nephrology, Massachusetts General Hospital, Boston, Massachusetts, USA

**Keywords:** acute kidney injury, COVID-19, influenza, mortality

## Abstract

**Introduction:**

Acute kidney injury (AKI) is a common complication in patients with severe COVID-19. We sought to compare the AKI incidence and outcomes among patients hospitalized with COVID-19 and with influenza.

**Methods:**

This was a retrospective cohort study of patients with COVID-19 hospitalized between March and May 2020 and historical controls hospitalized with influenza A or B between January 2017 and December 2019 within a large health care system. Cox proportional hazards models were used to compare the risk of AKI during hospitalization. Secondary outcomes included AKI recovery, mortality, new-onset chronic kidney disease (CKD), and ≥25% estimated glomerular filtration rate (eGFR) decline.

**Results:**

A total of 2425 patients were included; 1091 (45%) had COVID-19, and 1334 (55%) had influenza. The overall AKI rate was 23% and 13% in patients with COVID-19 and influenza, respectively. Compared with influenza, hospitalized patients with COVID-19 had an increased risk of developing AKI (adjusted hazard ratio [aHR] = 1.58; 95% confidence interval [CI], 1.29–1.94). Patients with AKI were more likely to die in the hospital when infected with COVID-19 versus influenza (aHR = 3.55; 95% CI, 2.11–5.97). Among patients surviving to hospital discharge, the rate of AKI recovery was lower in patients with COVID-19 (aHR = 0.47; 95% CI, 0.36–0.62); however, among patients followed for ≥90 days, new-onset CKD (aHR = 1.24; 95% CI, 0.86–1.78) and ≥25% eGFR decline at the last follow-up (aHR = 1.36, 95% CI, 0.97–1.90) were not significantly different between the cohorts.

**Conclusion:**

AKI and mortality rates are significantly higher in patients with COVID-19 than influenza; however, kidney recovery among long-term survivors appears to be similar.

## Graphical abstract

**Figure:**
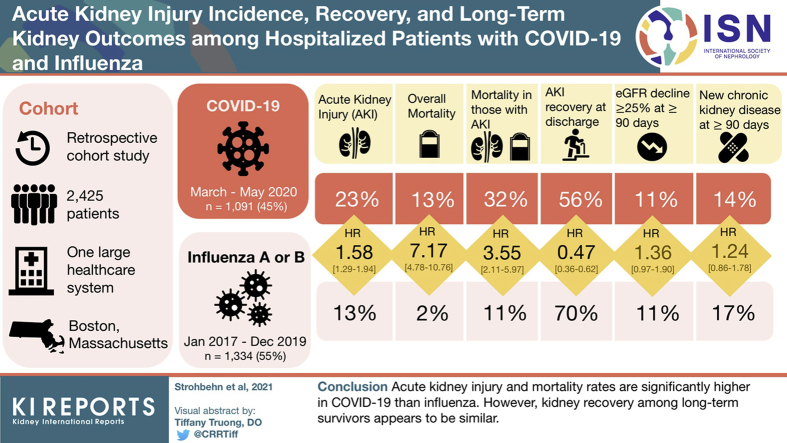

There have been nearly 150 million cases of COVID-19 worldwide as of the end of April 2021.^1^ COVID-19 is a respiratory illness that ranges in severity from mild upper respiratory symptoms to respiratory failure and death. Severe COVID-19 can cause multiorgan injury,^2^^,^^3^ and AKI has emerged as a common complication of COVID-19 among hospitalized patients, affecting between 17% and 37%.^4^, ^5^, ^6^, ^7^, ^8^, ^9^ AKI is associated with a 9-fold increased risk of death during a hospital stay.^10^

In the earliest autopsy series evaluating kidney histopathology in 26 patients who died of COVID-19 in China, the most common finding was prominent acute tubular necrosis, with a considerable number of patients also displaying endothelial injury and obstruction of peritubular and glomerular capillary lumens with erythrocytes.^3^ Various studies have also demonstrated elevated rates of dipstick-positive proteinuria and hematuria, and several glomerular diseases have been reported, including collapsing focal segmental glomerulosclerosis in patients with high-risk *APOL1* risk alleles.^4^^,^^11^, ^12^, ^13^, ^14^, ^15^, ^16^ Significant subclinical kidney damage may occur even in those who do not have AKI; kidney damage was discovered in autopsies of patients who did not have elevated creatinine at death.^3^ Thus, it has been theorized that patients infected with COVID-19 might be at risk of developing CKD.

There has been debate over whether AKI rates are higher in COVID-19 compared with other forms of sepsis, including influenza and community-acquired pneumonia.^17^ AKI may also complicate cases of severe influenza; autopsy series have also shown acute tubular necrosis as the dominant kidney lesion in fatal cases of influenza.^4^^,^^16^^,^^18^, ^19^, ^20^, ^21^, ^22^, ^23^, ^24^, ^25^, ^26^, ^27^, ^28^, ^29^, ^30^, ^31^, ^32^, ^33^, ^34^, ^35^, ^36^ We sought to compare the risk of AKI in hospitalized patients with COVID-19 compared with contemporary historical controls admitted with influenza. Additionally, among those surviving to hospital discharge, we sought to compare the rate of AKI recovery and new-onset CKD.

## Methods

### Patient Population and Data Acquisition

We conducted an observational, retrospective cohort study of hospitalized adults with COVID-19 within the Mass General Brigham health care system located in the Boston region. Our institutional review board approved the protocol and waived the need for informed consent. Research was conducted in accordance with the Helsinki Declaration. We obtained clinical data from the Research Patient Data Registry, which is the central data repository of Mass General Brigham used for research and quality improvement purposes.^37^^,^^38^ We included consecutive individuals infected with COVID-19 confirmed by real-time quantitative polymerase chain reaction between March 5, 2020, and May 31, 2020, who were hospitalized within 2 weeks of their first positive test. For our comparison group, we included consecutive individuals who were hospitalized within 2 weeks of a positive influenza A or B antigen or polymerase chain reaction test from January 1, 2017 to December 30, 2019. Hospitalizations were defined by the presence of a hospital admission encounter lasting at least 24 hours.

We excluded those who did not have at least 1 outpatient serum creatinine measured between 14 and 365 days before their first positive COVID-19 or influenza test in order to reduce any misclassification bias. We excluded patients with a baseline eGFR <15 ml/min per 1.73 m^2^ and those on dialysis. Patients were followed until death or 10 months after a diagnosis of COVID-19 or influenza; they were censored at the time of death or their last serum creatinine value recorded.

### Study Definitions

Baseline demographics were obtained from the Research Patient Data Registry demographics domain. Race/ethnicity was defined by incorporating race data and the primary language spoken using an algorithm shown in Supplementary Table S1. Baseline creatinine was defined by the outpatient serum creatinine value closest to hospitalization occurring between 14 and 365 days before the first positive COVID-19 or influenza test. The baseline eGFR was calculated using the CKD Epidemiology Collaboration equation.^39^ Comorbidities were defined by the presence of at least 2 diagnosis codes using the diagnoses domain (Supplementary Table S2). Baseline medications were defined by at least 1 instance of a prescribed medication within 1 year of baseline obtained from the medications domain (Supplementary Table S3). Laboratory studies at the time of admission were defined as those performed on the day of admission. If laboratory studies were not available on the day of admission, the next closest laboratory value within 72 hours before or 24 hours after admission was recorded. Missing data for admission laboratory studies are shown in Supplementary Table S4.

### Outcomes

The primary outcome was the incidence of AKI during hospitalization, which was defined as a 1.5-fold or greater rise in serum creatinine from baseline. AKI stage 2 (defined as a ≥2-fold increase or greater in creatinine from baseline) and AKI stage 3 (defined as a ≥3-fold increase or greater) were secondary outcomes. For the purposes of our models and analyses, AKI stages are presented as being inclusive of higher stages (e.g., AKI stage 1 includes AKI stage 1 or higher). Incident dialysis was not captured in the Research Patient Data Registry data set. Additional outcomes included (1) recovery of serum creatinine to within 20% of baseline creatinine by hospital discharge as defined by Siew *et al.*^40^ and (2) mortality during the hospital stay among patients with AKI and the overall cohort. Among patients who survived hospital discharge and whose kidney function was followed for at least 90 days, we determined (1) new-onset CKD defined by an eGFR <60 ml/min per 1.73 m^2^ separated by at least 90 days occurring in a patient whose prehospitalization baseline eGFR was ≥60 ml/min per 1.73 m^2^ and (2) the development of ≥25% glomerular filtration rate decline at the last follow-up compared with the prehospital baseline eGFR.

### Statistical Analysis

Baseline characteristics were described using means and SDs for normally distributed data and medians and interquartile ranges (IQR) for non-normally distributed data. Counts and percentages were used for categoric variables. Univariate and multivariable Cox proportional hazards models were performed to estimate the hazard ratios of each outcome (AKI, AKI recovery by hospital discharge, in-hospital mortality, incidence of new-onset CKD, and ≥25% eGFR decline) by comparing the COVID-19 and influenza cohorts. Covariates considered for confounding adjustment included age, sex, race, baseline creatinine, hypertension, diabetes mellitus, cirrhosis, angiotensin-converting enzyme inhibitors/angiotensin II receptor blockers, diuretics, and proton pump inhibitors.

Sensitivity analyses were performed to assess the robustness of our findings using propensity score adjustment by (1) matching patients with COVID-19 to those with influenza based on their propensity score and (2) inverse probability treatment weighting.^41^^,^^42^ Propensity scores were determined based on a multivariable logistic regression model that estimated the probability of having a COVID-19 versus influenza hospital admission. The covariates in the logistic regression model were age, sex, race/ethnicity, baseline creatinine, diabetes mellitus, chronic obstructive pulmonary disease, congestive heart failure, cirrhosis, human immunodeficiency virus, coronary artery disease, angiotensin-converting enzyme inhibitors/angiotensin II receptor blockers, loop diuretics, thiazide diuretics, potassium-sparing diuretics, proton pump inhibitors, nonsteroidal anti-inflammatory drugs, and immunosuppressants. We then matched the COVID-19 patients with those with influenza using 1:1 nearest neighbor greedy matching without replacement and a caliper of 0.2 SD of the propensity score. Standardized differences were calculated between the 2 cohorts in the original population and after matching or weighting.^41^^,^^43^ The relative risk of developing AKI for COVID-19 patients was estimated by performing Cox regression stratified on matched pairs for the matched cohorts and adjusted for inverse probability treatment weighting, respectively.

Statistical analyses were performed using R Version 1.1.463 (R Foundation for Statistical Computing, Vienna, Austria) and SAS Version 16 (SAS Institute, Cary, NC). All *P* values were 2-sided, and *P* < 0.05 was considered significant.

## Results

### Cohorts and Study Timeline

**Figure 1:**
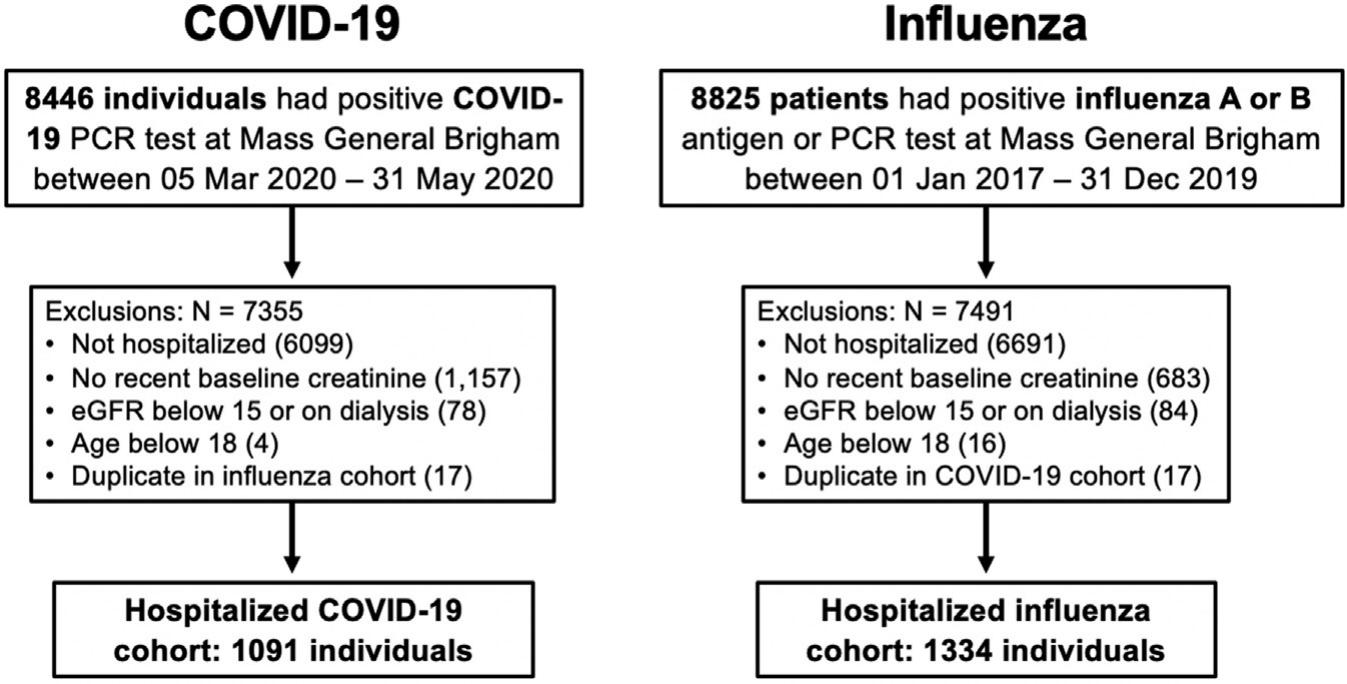
Patient flow and study exclusions. eGFR, estimated glomerular filtration rate; PCR, polymerase chain reaction.

There were 8446 individuals who tested positive for COVID-19 between March 5, 2020, and May 31, 2020. After applying the exclusions shown in Figure 1, we included 1091 hospitalized individuals infected with COVID-19 with an established baseline eGFR ≥15 ml/min per 1.73 m^2^. There were 8825 individuals who tested positive for influenza between January 1, 2017, and December 30, 2019. After applying the exclusions shown in Figure 1, we included 1334 hospitalized individuals with influenza. There were 871 (80%) patients with COVID-19 and 1230 (92%) patients with influenza who were hospitalized on the day they tested positive; of those who were hospitalized in the days after a positive test (220 with COVID-19 and 104 with influenza), the median time between diagnosis and hospital admission was 4 days (IQR, 2–7 days) for 220 patients with COVID-19 and 6 days (IQR, 2–9 days) for patients with influenza. The mean length of stay is 9.9 days (SD = 10.8 days) for COVID-19 and 5.7 days (SD = 5.7 days) for influenza. The mean time from diagnosis to the last creatinine value was an average of 117 days (SD = 113 days) for patients with COVID-19 and 174 days (SD = 110 days) for patients with influenza.

### Baseline Characteristics

Baseline characteristics are shown in Table 1. Age, sex, and baseline creatinine were similar across both cohorts. Race and ethnicity differed between the 2 groups; in the COVID-19 cohort, 53% were White, 22% were Hispanic, and 17% were Black, and in the influenza cohort, 76% were White, 8% were Hispanic, and 9% were Black. The baseline eGFR was higher in the COVID-19 cohort than in the influenza cohort (76 ml/min per 1.73 m^2^ vs. 70 ml/min per 1.73 m^2^). Comorbidities were more common in patients hospitalized with influenza compared with COVID-19, with a higher prevalence of congestive heart failure (20% vs. 10%), coronary artery disease (59% vs 41%), and chronic obstructive pulmonary disease (40% vs. 16%) than in the influenza cohort. Medication use was similar in the 2 cohorts, with the main difference being higher use of immunosuppressant medications in patients with influenza (39% in influenza vs. 17% in COVID-19). Standardized differences between baseline characteristics in nonpropensity score matching, propensity score matching, and inverse probability treatment weighting cohorts are shown in Supplementary Table S5.

**Table 1:** Baseline characteristics

	COVID-19 (*n* = 1091)	Influenza (*n* = 1334)
Age (yr)	67 (18)	71 (16)
Sex, male, n (%)	540 (50)	599 (45)
Baseline creatinine, mean (SD)	1.02 (0.41)	1.06 (0.44)
Baseline eGFR (mean (SD))	75.9 (27.7)	69.6 (26.2)
eGFR stages (n (%)		
eGFR ≥60	760 (70)	811 (61)
eGFR 45–59.9	160 (15)	263 (20)
eGFR 30–44.9	123 (11)	180 (13)
eGFR 15–29.9	48 (4)	80 (6)
Race/ethnicity, n (%)		
White, non-Hispanic	578 (53)	1018 (76)
White, Hispanic	244 (22)	100 (8)
Black^a^	184 (17)	119 (9)
Other/unknown	85 (8)	97 (9)
Comorbidities, n (%)		
Hypertension	839 (77)	1136 (85)
Diabetes mellitus	549 (50)	602 (45)
Coronary artery disease	443 (41)	783 (59)
Chronic obstructive pulmonary disease	175 (16)	528 (40)
Congestive heart failure	113 (10)	271 (20)
Cirrhosis	53 (5)	80 (6)
Human immunodeficiency virus	24 (2)	32 (2)
Medications, n (%)		
ACEi/ARBs	397 (36)	535 (40)
Loop diuretics	280 (26)	467 (35)
Thiazide diuretics	162 (15)	198 (15)
K-sparing diuretics	63 (6)	116 (9)
Proton pump inhibitors	381 (35)	615 (46)
Nonsteroidal anti-inflammatory drugs	371 (34)	425 (32)
Immunosuppressants	185 (17)	521 (39)
Baseline laboratory values, median (IQR)		
Hemoglobin, g/dl	12.5 (11.2–13.8)	12.3 (10.8–13.6)
White blood cell count, K/μl	7.35 (5.91–9.32)	7.55 (5.87–9.62)
Sodium, mmol/l	140 (138–142)	140 (138–142)
Platelets, K/μl	227 (184–278)	219 (174–279)
Albumin, g/dl	4.1 (3.7–4.4)	4.0 (3.6–4.3)

ACEi, angiotensin-converting enzyme inhibitor; ARB, angiotensin II receptor blocker; eGFR, estimated glomerular filtration rate; IQR, interquartile range.

Missing data: hemoglobin (COVID-19 = 127 [12%], influenza = 86 [7%]), white blood cells (COVID-19 = 128 [12%], influenza = 88 [7%]), sodium (COVID-19 = 20 [2%], influenza = 14 [1%]), platelets (COVID-19 = 130 [12%], influenza = 88 [7%]), and albumin (COVID-19 = 275 [25%], influenza = 287 [22%]).

a Individuals who were listed as both Black and Hispanic were classified as Black for the purposes of our analyses.

Admission laboratory results among patients with AKI are shown in Table 2. We did not detect major differences between admission laboratory values between patients with COVID-19 and influenza. Inflammatory and coagulation markers such as C-reactive protein, interleukin-6, and D-dimer were elevated in patients with COVID-19; however, they were not routinely measured for patients with influenza, limiting any comparisons (Supplementary Table S6).

**Table 2:** Admission laboratory values for patients with acute kidney injury

Laboratory test, units	COVID-19 (*n* = 251) median (IQR)	Influenza (*n* = 179) median (IQR)
WBC count, K/μl	7.38 (5.45–9.66)	8.05 (5.85–11.30)
Hemoglobin,^a^ g/dl	12.5 (10.7–13.9)	11.4 (10.1–13.4)
Hematocrit,^a^ %	38.2 (33.9–42.5)	35.3 (30.5–40.6)
Platelets, K/μl	179 (134–235)	182 (128–240)
Sodium, mmol/l	137 (133–140)	137 (133–139)
Potassium, mmol/l	4.2 (3.8–4.5)	4.0 (3.7–4.5)
Chloride,^a^ mmol/l	98 (94–102)	97 (93–100)
Bicarbonate,^a^ mmol/l	22 (19–24)	24 (20–27)
BUN, mg/dl	27 (17–45)	24 (16–36)
Creatinine, mg/dl	1.31 (0.95–2.08)	1.41 (1.05–1.89)
Albumin, g/dl	3.5 (3.0–3.8)	3.6 (3.0–4.0)
Neutrophils,^a^ %	77.0 (69.7–83.1)	74.5 (66.0–84.0)
ALT, U/l	26 (16–39)	24 (15–42)
AST, U/l	44 (28–64)	33 (24–64)
Total bilirubin, mg/dl	0.5 (0.3–0.7)	0.5 (0.3–0.8)
Lactate, mmol/l	1.6 (1.1–2.6)	1.6 (1.2–2.0)
PT-INR, NA	1.1 (1.1–1.3)	1.2 (1.1–1.8)
PTT, s	37 (33–46)	39 (33–58)

ALT, alanine aminotransferase; AST, aspartate aminotransferase; BUN, blood urea nitrogen; NA, not applicable; PT-INR, prothrombin time and international normalized ratio; PTT, partial thromboplastin time; WBC, white blood cell.

Laboratory studies at the time of admission were defined as those performed on the day of admission; if laboratory studies were not available on the day of admission, the next closest laboratory value within 72 hours before or 24 hours after admission was recorded.

a Represents laboratory values that were significantly different between COVID-19 and influenza cohorts with a *P* value < 0.05. Supplementary Table S4 shows the missing data for the time of hospitalization laboratory findings.

### AKI Incidence

**Figure 2:**
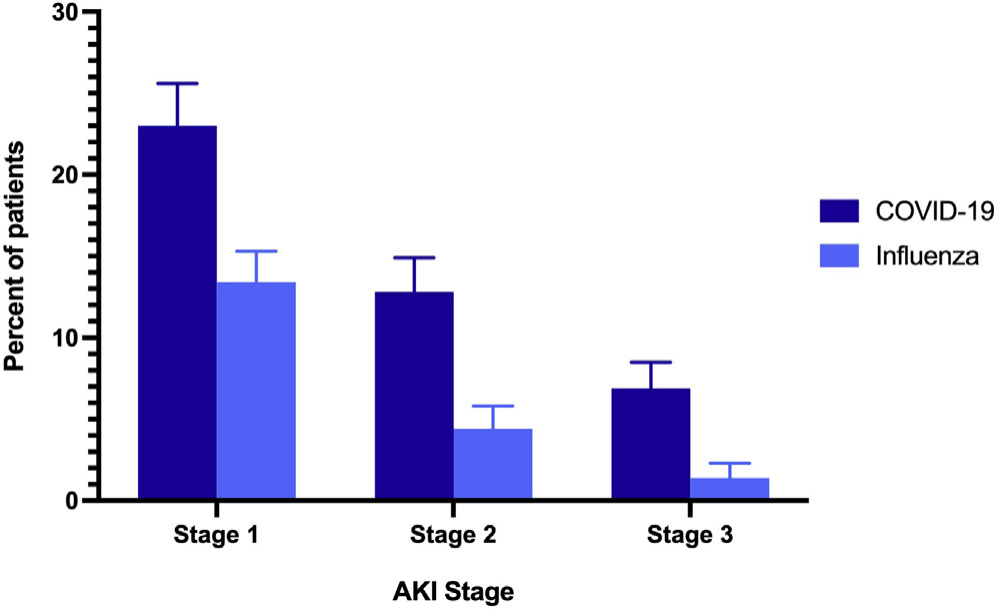
The percent of hospitalized patients with acute kidney injury (AKI). AKI stages are inclusive of higher stages (e.g., AKI stage 1 includes AKI stage 1, stage 2, and stage 3 patients).

**Figure 3:**
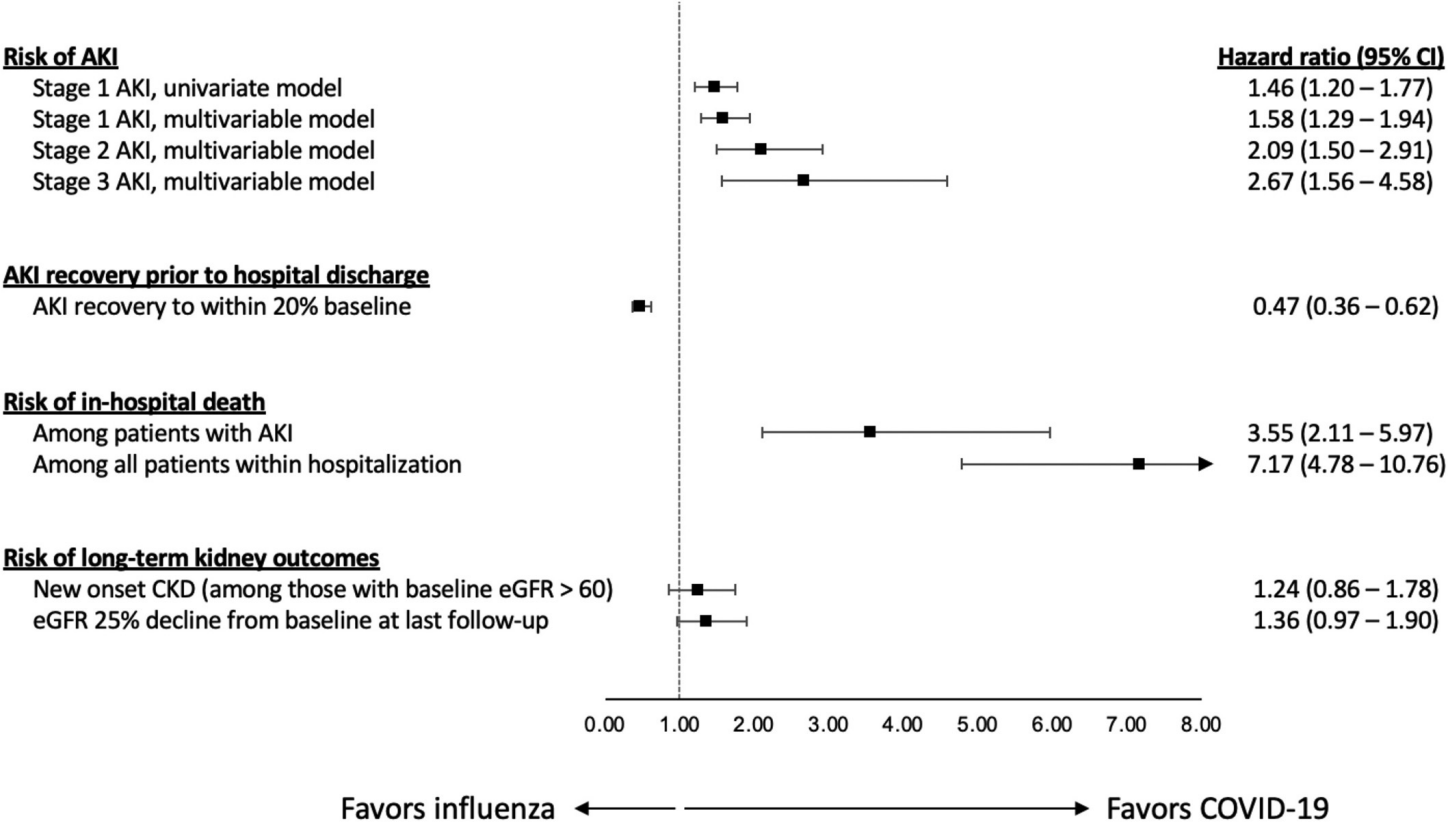
The association between COVID-19 versus influenza and kidney outcomes. All models were adjusted for the following covariates: age, sex, baseline creatinine, race/ethnicity (White/Hispanic/Black/other), and COVID-19 versus influenza. Models are shown in Supplementary Table S7A–G and Supplementary Table 8A and B. AKI, acute kidney injury; CI, confidence interval; CKD, chronic kidney disease; HR, hazard ratio. 217 × 119 mm (300 × 300 dots per inch).

The incidence and severity of AKI in patients hospitalized with COVID-19 and influenza are shown in Figure 2. Unadjusted overall AKI stage 1 or higher rates were 251 (23%) in the COVID-19 cohort and 179 (13%) in the influenza cohort (*P* < 0.01), AKI stage 2 or higher rates were 140 (13%) in the COVID-19 cohort and 58 (4%) in the influenza cohort (*P* < 0.01), and AKI stage 3 rates were 75 (7%) in the COVID-19 cohort and 19 (1%) in the influenza cohort (*P* < 0.01). The results of the univariate and multivariable models comparing the incidence of AKI between influenza and COVID-19 are shown in Figure 3. The median time to the first occurrence of AKI was 6 days (IQR, 3–10 days) and 4 days (IQR, 2–6 days) after baseline, respectively. The unadjusted hazard ratio for AKI stage 1 or higher was 1.46 (95% CI, 1.20–1.77) (Figure 3, Table 3). After adjusting for confounding factors, the risk of AKI was higher in patients with COVID-19 compared with patients with influenza; the estimated aHRs for AKI stage 1 or higher, stage 2 or higher, and stage 3 were 1.58 (95% CI, 1.29–1.94), 2.09 (95% CI, 1.50–2.91), and 2.67 (95% CI, 1.56–4.58), respectively (Figure 3, Table 3, Supplementary Tables S7A and S7B). Consistent findings were observed from sensitivity analyses using propensity score approaches (Supplementary Tables S8A and S8B).

**Table 3:** Acute kidney injury stage 1 or higher among hospitalized patients (univariable and multivariable Cox proportional hazards model)

Variable	Unadjusted HR	95% CI	*P* value	Multivariable aHR	95% CI	*P* value
Age	1.00	1.00–1.01	0.99	0.99	0.99–1.00	0.06
Sex, male	1.12	0.93–1.35	0.25	1.07	0.88–1.31	0.49
Baseline creatinine	1.14	0.92–1.41	0.22	0.88	0.69–1.12	0.30
Race						
White	**0.72**	**0.55–0.93**	**0.01**	0.83	0.63–1.09	0.18
Hispanic	0.74	0.52–1.03	0.08	**0.67**	**0.47–0.95**	**0.02**
Black	—	—	—	—	—	—
Other	0.68	0.44–1.05	0.08	0.72	0.47–1.12	0.15
COVID-19 vs. influenza	**1.46**	**1.20–1.77**	**<0.01**	**1.58**	**1.29–1.94**	**<0.01**
Comorbidities						
Hypertension	**2.11**	**1.54–2.89**	**<0.01**	**1.77**	**1.22–2.55**	**<0.01**
Diabetes mellitus	**1.57**	**1.29–1.90**	**<0.01**	**1.29**	**1.05–1.58**	**0.01**
COPD	1.05	0.85–1.29	0.68	—	—	—
CHF	1.20	0.94–1.53	0.16	—	—	—
Cirrhosis	**1.72**	**1.23–2.41**	**<0.01**	**1.47**	**1.03–2.09**	**0.03**
HIV	0.80	0.40–1.60	0.53	—	—	—
CAD	1.10	0.91–1.33	0.32	—	—	—
Medications						
ACEi/ARBs	**1.71**	**1.41–2.06**	**<0.01**	**1.42**	**1.15–1.75**	**<0.01**
Loop diuretics	**1.43**	**1.18–1.74**	**<0.01**	**1.26**	**1.01–1.57**	**0.04**
Thiazide diuretics	**1.43**	**1.13–1.81**	**<0.01**	1.08	0.84–1.39	0.55
K-sparing diuretics	**1.90**	**1.42–2.54**	**<0.01**	**1.47**	**1.07–2.01**	**0.02**
PPIs	1.14	0.94–1.38	0.18	—	—	—
NSAIDs	0.90	0.73–1.10	0.29	—	—	—
Immunosuppressants	1.18	0.96–1.45	0.11	—	—	—

ACEi, angiotensin-converting enzyme inhibitor; aHR, adjusted hazard ratio; ARB, angiotensin II receptor blocker; CAD, coronary artery disease; CHF, congestive heart failure; CI, confidence interval; COPD, chronic obstructive pulmonary disease; HR, hazard ratio; NSAID, nonsteroidal anti-inflammatory drug; PPI, proton pump inhibitor.

Variables with *P* < 0.05 in the univariable model are included in the multivariable model; all statistically significant values are bolded.

### Mortality During Hospital Stay

The overall mortality rate during the hospital stay was 145 (13%) patients with COVID-19 and 31 (2%) patients with influenza (*P* < 0.01); the aHR for death was 7.17 (95% CI, 4.78−10.76) (Figure 3, Supplementary Table S7C).

**Figure 4:**
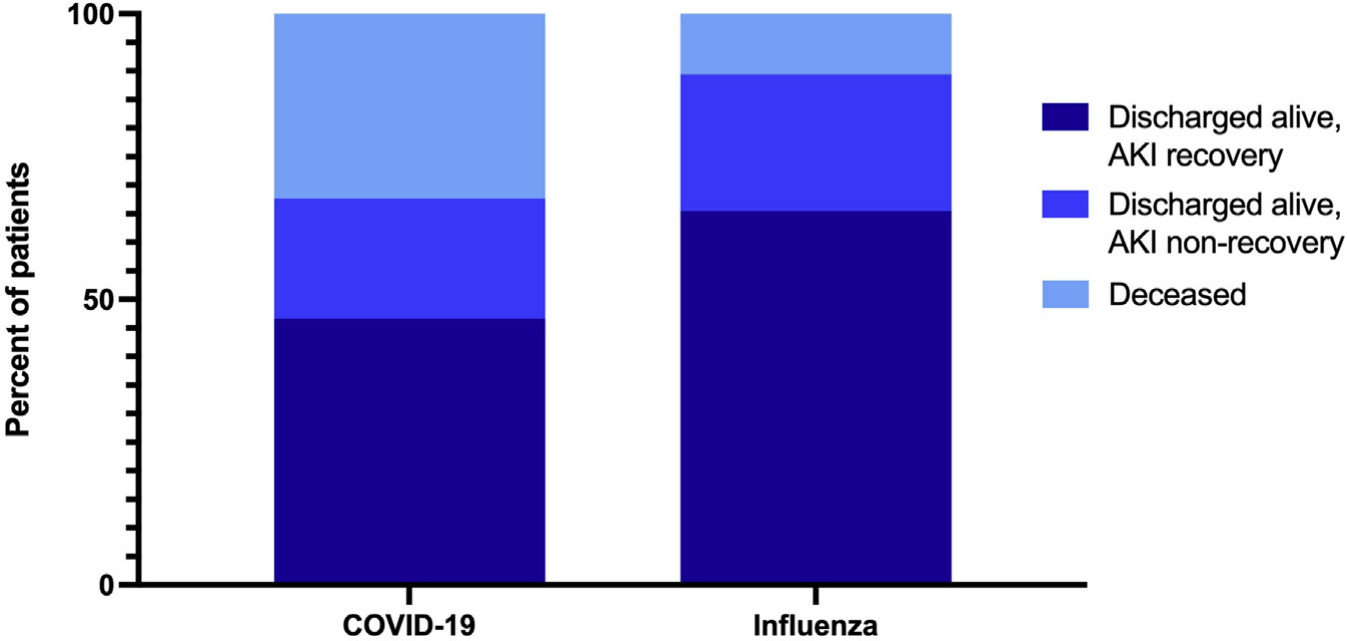
Hospital outcomes among patients with acute kidney injury (AKI).

In-hospital outcomes among patients with AKI are shown in Figure 4. Compared with AKI patients with influenza, those in the COVID-19 cohort had a higher rate of in-hospital mortality (81/251 [32%] vs. 19/179 [11%], *P* < 0.01). After adjusting for confounders at baseline, the aHR for in-hospital mortality was 3.55 (95% CI, 2.11–5.97) for patients with COVID-19−associated AKI versus influenza-associated AKI (Figure 3, Supplementary Table S7D).

### Kidney Recovery and Long-term Kidney Outcomes Among Survivors

AKI recovery rates were lower in patients with COVID-19. Among the 251 patients with COVID-19−associated AKI, 140 (56%) recovered to within 20% of baseline creatinine, whereas among the 179 patients with influenza-associated AKI, 126 (70%) recovered to within 20% of baseline creatinine (*P* < 0.01). In the multivariable model, the aHR for AKI recovery before hospital discharge was 0.47 (95% CI, 0.36–0.62) for patients with COVID-19 versus influenza (Figure 3, Supplementary Table S7E).

We then examined long-term renal outcomes in patients who survived to discharge and were followed for at least 90 days. Ten months had elapsed since the diagnosis of COVID-19 in all of the included patients, so we censored follow-up at 10 months after baseline in all participants. Among patients with COVID-19, 525 (48%) survived to hospital discharge and were followed for ≥90 days. Among these patients, 60 (11%) had an eGFR decline ≥25% from baseline. Among patients with influenza, 947 (71%) survived to hospital discharge and were followed for ≥90 days. Among these patients, 103 (11%) had an eGFR decline ≥25% from baseline (*P* = 0.81). Patients with COVID-19 had an insignificant trend toward a higher risk of a long-term eGFR decline of ≥25% (aHR = 1.36; 95% CI, 0.97–1.90) compared with those with influenza (Figure 3, Supplementary Table S7F).

We also determined the risk of new-onset CKD (defined as an eGFR <60 ml/min per 1.73 m^2^ separated by more than 90 days) in patients who did not have CKD at baseline (Table 3). Among survivors, there were 51 (14%) cases of new-onset CKD among 374 in the COVID-19 cohort and 95 (17%) cases among 575 in the influenza cohort (*P* = 0.27). In the multivariable model, the aHR for new-onset CKD during the 10-month follow-up was 1.24 (95% CI, 0.86–1.78) for patients with COVID-19 versus influenza (Figure 3, Supplementary Table S7G).

## Discussion

In our study examining AKI incidence and outcomes among hospitalized patients with COVID-19 compared with a historical control group of patients who were hospitalized with influenza between 2017 and 2019 in a large health care system, we found that patients hospitalized due to COVID-19 were 1.58 times more likely to develop AKI compared with patients with influenza. For AKI stage 2 or higher and AKI stage 3, there was a 2.09-fold and 2.67-fold higher risk, respectively, in patients with COVID-19 compared with those with influenza. Our findings were robust because similar findings were observed from the sensitivity analyses using propensity score approaches. Additionally, we found a dramatically higher risk of overall mortality during the hospital stay and a higher mortality risk among patients with COVID-19−associated AKI compared with influenza-associated AKI. Patients with COVID-19−associated AKI were less likely to recover before hospital discharge; however, among patients who survived to hospital discharge and were followed for ≥90 days, the rates of progressive eGFR decline ≥25% from baseline and new-onset CKD were not significantly different. Our study is among the first articles to provide estimates of AKI recovery that extend beyond the index hospitalization as well as providing long-term follow-up (10 months post infection).

Our study highlights the difference between AKI in COVID-19 and influenza and adds to a growing body of literature that sheds light on the magnitude of the effect of COVID-19 on kidney function. In our study, 23% of hospitalized patients with COVID-19 experienced AKI, which is within the range found in other studies of AKI rates in hospitalized patients in the United States, estimated to be between 17% and 37%.^4^, ^5^, ^6^, ^7^, ^8^, ^9^ Robbins-Juarez *et al.*^9^ conducted a meta-analysis of 20 studies consisting of 13,137 hospitalized patients in total and found that AKI occurred in 17%, with a range of 0.5% to 80.3%. Our cohort was more than 10 years older on average and tended to have more comorbidities compared with the patients in this prior meta-analysis, with higher rates of baseline CKD and other comorbidities. Fisher *et al.*^44^ compared AKI rates in patients hospitalized with COVID-19 in another New York City area hospital system and found a 2.3 (95% CI, 2.2–2.4) adjusted risk of AKI compared with a mix of hospitalized patients from the same hospital system 1 year earlier, defining AKI as a 0.3 mg/dl increase or >50% increase in serum creatinine from the baseline creatinine to the maximum creatinine during the hospital stay. Using patients with a similar reason for admission (influenza) makes our control group more homogenous and highlights the uniquely high risk associated with COVID-19 compared with another potentially life-threatening viral respiratory infection. Piroth *et al.*^45^ used the French national administrative database to compare outcomes in hospitalized patients with COVID-19 or influenza during the same time frame. They found an AKI rate of 6.4% among patients with COVID-19 and 4.9% among patients with influenza, which was statistically significant. Of note, in this study, AKI was defined by diagnosis codes, which likely explains the lower incidence. Xie *et al.*^7^ recently compared outcomes among hospitalized patients diagnosed with COVID-19 and influenza in the US Department of Veterans Affairs Database. They also found that AKI (defined as either a 0.3-mg/dl increase or ≥1.5 times baseline creatinine) was higher in COVID-19 than influenza, estimating a 1.52 (95% CI, 1.37–1.69) increased risk. Our study expands on this by demonstrating that the risk of higher stages of AKI is even more dramatically increased in patients with COVID-19. Additionally, we revealed a higher risk of mortality by the end of the hospital stay among patients with COVID-19 with AKI compared with patients with influenza who had AKI. Finally, because our study only included patients with known baseline creatinine within 14 to 365 days before admission, we were able to assess AKI recovery and long-term kidney outcomes.

There is no standardized definition for AKI recovery. Using a stricter definition than other recently published series in patients with COVID-19, we found that 56% of patients with COVID-19 recovered to within 20% of prehospital baseline by discharge, which was significantly lower than in patients with influenza. Ng *et al.*^46^ reported that 74% of hospitalized patients with COVID-19 had an improved serum creatinine by the time of discharge, which was defined as a decline of 33% in the discharge serum creatinine level from the peak admission serum creatinine level. Stevens *et al.*^8^ reported that 41% of patients with COVID-19 who had received renal replacement therapy for AKI achieved recovery, defined by cessation of dialysis treatment before discharge. Gupta *et al*.^47^ studied patients hospitalized with COVID-19 in the intensive care unit who had received renal replacement therapy for AKI and found that 143 (66%) were able to discontinue dialysis by discharge. Stockmann et al.^48^ revealed that for patients with dialysis-dependent AKI who survived to discharge, 92% had discontinued dialysis, and 62% had a full recovery of kidney function at a median of 151 days.

To date, there are no large reports of long-term kidney outcomes in patients who develop COVID-19. In our study, we found similar rates of new onset CKD or ≥25% eGFR decline between the COVID-19 and influenza cohorts in survivors followed for up to 10 months after diagnosis of COVID-19 or influenza. This result is limited by the fact that approximately 50% of surviving patients did not have a creatinine measured ≥90 days after a diagnosis of COVID-19; thus, there is a large effect of missing data that may bias our estimates. Prospective studies that measure kidney function via blood and urine tests in large cohorts with COVID-19 will be needed to understand the true burden of kidney disease. Our study highlights the importance of close, postdischarge follow-up for patients with AKI.

Our study has several additional limitations. This is a single health care system and therefore limits generalizability. However, the COVID-19 cohort of patients was racially and ethnically diverse. As prior studies have noted, the burden of COVID-19 on minority populations is reflected by the fact that a substantially larger proportion of Hispanic and Black individuals were hospitalized with COVID-19 compared with the historical controls who were hospitalized with influenza in our health care system.^49^ Our study was unable to directly compare certain inflammatory markers between COVID-19 and influenza. Patients with COVID-19 had elevated markers of inflammation and blood coagulation, but we could not make a direct comparison to influenza because these markers were so infrequently checked in patients with influenza (Supplementary Table S6). We could not reliably capture urine output or renal replacement in our electronic database and thus could not use this criteria for our AKI definition, which was based only on fold-creatinine changes. Because we only included patients who had a baseline creatinine, this ultimately led to the exclusion of considerable numbers of hospitalized patients (Figure 1); however, this was necessary in order to accurately define AKI recovery and determine the rate of new-onset CKD. Additionally, by capturing a single baseline creatinine within 1 year of baseline, it meant that some patients had different follow-up intervals compared with others. Finally, our electronic health record database did not capture important markers of the acuity of illness, including the use of vasopressors, mechanical ventilation, incident dialysis, and intensive care unit stay; thus, we could not correct for severity among hospitalized patients. Nonetheless, we believe that disease severity among hospitalized patients may be a mediator of differential rates of AKI, and, therefore, our primary analysis is valuable for providers caring for these patients. Studies that evaluate illness severity markers on long-term kidney function in patients with COVID-19 will be needed.

Our study confirms that the risk of AKI from COVID-19 exceeds the risk of AKI with influenza in patients who are hospitalized. The risk of AKI stage 2 or higher is greater than 2-fold in patients with COVID-19 than patients with influenza; additionally, those with COVID-19 who suffer AKI are 3.6 times more likely to die and are nearly half as likely to recover by hospital discharge. However, among patients with COVID-19 who survive to hospital discharge and are followed for ≥90 days, there does not appear to be a heightened risk of new-onset CKD nor a ≥25% eGFR decline within 10 months of baseline. Larger studies of inpatients and outpatients with longer follow-up will be needed to define long-term risks of COVID-19 on kidney function.

## Disclosure

MES has received research grants to institution and has served as a scientific advisory board member to Gilead. ASA has received research grants from the American Heart Association and from Mallinckrodt Pharmaceuticals, and has served as a scientific advisory board member to Mallinckrodt Pharmaceuticals. All the other authors declared no competing interests.

## Acknowledgments

MES was supported by 10.13039/100000002NIH R03 DK 128553 and K23 DK 117014.

## Supplementary Material

Supplementary File (PDF)

**Table S1.** Race code definitions.

**Table S2.** Diagnosis codes.

**Table S3.** Medication list.

**Table S4.** Missing laboratory values at hospital admission.

**Table S5.** Standardized differences between baseline characteristics in nonpropensity score matching, propensity score matching, and inverse probability treatment weighting cohorts.

**Table S6.** Comparison of additional laboratory tests in patients with COVID-19 and influenza related acute kidney injury.

**Table S7A.** Acute kidney injury stage 2 or higher among hospitalized patients (multivariable Cox proportional hazards model).

**Table S7B.** Acute kidney injury stage 3 among hospitalized patients (multivariable Cox proportional hazards model).

**Table S7C.** In-hospital mortality among the total cohort (multivariable Cox proportional hazards model).

**Table S7D.** In-hospital mortality among patients with acute kidney injury stage 1 or higher (multivariable Cox proportional hazards model).

**Table S7E.** Acute kidney injury recovery to within 20% of prehospital baseline creatinine by hospital discharge (multivariable Cox proportional hazards model).

**Table S7F.** Estimated glomerular filtration rate decline ≥25% at last follow-up among patients followed for ≥90 days (multivariable Cox proportional hazards model).

**Table S7G.** New-onset chronic kidney disease among patients followed for ≥90 days (multivariable Cox proportional hazards model).

**Table S8A.** Acute kidney injury stage 1 or higher among hospitalized patients (multivariable Cox proportional hazards model, propensity score cohort).

**Table S8B.** Acute kidney injury stage 1 or higher among hospitalized patients (multivariable Cox proportional hazards model, propensity score inverse probability treatment weighting cohort).
