# Supplementary material for: Acute Kidney Injury Incidence, Recovery, and Long-term Kidney Outcomes Among Hospitalized Patients With COVID-19 and Influenza
Source: Kidney Int Rep. 2021 Jul 15;6(10):2565–74. doi: 10.1016/j.ekir.2021.07.008 (PMC8280679; doi:10.1016/j.ekir.2021.07.008)
Supplement: Supplementary File (PDF) [file mmc1.pdf]

Supplemental Table S1. Race code definitions

Race	Codes
White	Race == "White-W" OR "White-WHITE"
Black	Race == "African American-AFRICAN AMERICAN" OR “Black-BLACK” OR “BLACK OR AFRICAN AMERICAN” OR “Hispanic Black-BLACK@HISPANIC” OR “Hispanic Black-BLACK OR AFRICAN AMERICAN@HISPANIC”<br><br>OR<br><br>Language == “Cape Verdean-CAPE VERDEAN” OR “Creole” OR “Haitian Creole” OR “French Creole-FRENCH CREOLE” OR “Somalian-SOMALI” OR “African Languages” OR “Amharic” OR “Ethiopian-Tigrinia-TIGRINYA (ETHIOPIA)” OR “Liberian-LIBERIAN” OR “Maay Maay” OR “Efik”
Hispanic	Race == “Hispanic-HISPANIC” OR “Asian-ASIAN@HISPANIC” OR “Dominican-DOMINICAN” OR “Dominican-DOMINICAN@HISPANIC” OR “Hispanic-SPANISH@HISPANIC” OR “Hispanic-H” OR “Hispanic-DECLINED@HISPANIC” OR “Hispanic-HISPANIC OR LATINO@HISPANIC” OR “hispanic-UNKNOWN@HISPANIC” OR “Hispanic-HISPANIC@HISPANIC” OR “Hispanic-@HISPANIC” OR “Hispanic-HISPANIC OR LATINO” OR “Hispanic-LATINO@HISPANIC” OR “Hispanic-OTHER@HISPANIC” OR “Hispanic-UNKNOWN@HISPANIC”<br><br>OR<br><br>Language == “Spanish-SPANISH” AND Race == “White-WHITE”<br><br>OR<br><br>Language == “Spanish-SPANISH” AND Race == “Not Recorded-UNKNOWN”<br><br>OR<br><br>Language == “Spanish-SPANISH” AND Race == “Other-OTHER”
Other	Race == “Asian-ASIAN” OR “Not Recorded-UNKNOWN” OR “Other-OTHER” OR “American Indian-AMERICAN INDIAN OR ALASKA NATIVE”

Supplemental Table S2. Diagnosis Codes

Diagnosis	Codes
Dialysis	Z99.2, V45.11
Hypertension	401.1, 401.9, 401.0, 459.31, 997.91, I10, I15.1, I15.9, I15.0, I87.311, I87.393, LPA196, MHAB1, LPA1305, HTN_pl0.5, 199, 134, I15.2, 405.01, I87.313, I15.8, I87.312, 405.99, 459.33, 459.30, I87.329, 405.91, I87.303, I87.309, I87.323, I87.333, I87.302, I87.332
Diabetes	250.00, 250.01, 249.20, 249.70, 250.02, 249.00, 250.10, 250.03, 250.42, 249.60, 249.80, 250.11, 249.11, 250.62, 250.80, 250.50, 250, 250.90, 250.92, 249.01, 250.60, 250.83, 250.40, 250.70, 250.81, 250.93, 250.20, 250.71, 250.61, 250.12, 250.13, 250.1, 250.52, 250.91, 250.51, 357.2, 648.00, E08.22, E10.319, E08.40, E10.3299, E10.65, E11.00, E11.10, E11.22, E11.29, E11.311, E11.319, E11.351, E11.3512, E11.3513, E11.39, E11.40, E11.42, E10.3293, E10.3393, E11.21, E11.3293, E10.36, E10.9, E10.10, E10.69, E10.8, E11.3511, E11.59, E11.621, E11.65, E10.339, E10.329, E11.649, E11.321, E11.51, E11.622, E11.49, E11.8, E11.9, E11.69, E13.10, E13.359, E13.39, E10.39, E11.329, E11.3599, E08.311, E10.3553, E11.3393, E11.3593, E11.43, E13.9, E11.3553, E13.3599, E08.9, E10.649, E11.3519, E13.01, E11.52, E10.349, E13.3593, E13.49, E11.3592, E13.65, E11.359, E11.36, E08.10, LPA105, LPA1059, LPA579, EHAT2, LPA1048, 294, 420, 250.72, 253.5, 250.73, 588.1, E11.3213, E11.3299, E23.2, E11.3313, LPA511, 250.0, 648.03, E08.00, E11.628, E13.621, E08.65, E08.621, E08.41, E11.3399, 250.82, 250.22, 648.01, E10.621, E11.638, E10.3512, E11.3211, E08.69, E08.8, E11.3291, O24.113, O24.119, O24.112, O24.12, O24.912, O24.313, E10.3211, LPA106, 250.43, E09.9, E10.42, E10.59, E08.29, E09.65, E10.49, E08.51, E11.01, E10.40, 250.41, 249.40, E10.51, E10.29, E11.641, E10.610, E11.3212, E11.3292, E11.11, O24.420, E08.01, E08.21, 250.63, E10.22, E10.3313, E13.319, E11.41, E08.351, E10.21, E11.3311, E11.3219, E13.622, O24.919, ELAC2, E13.22, E08.622, E08.3293, E08.319, 250.9, 249.61, E13.40, E08.3291, E11.3412, E10.11, E11.3552, N25.1, E11.610, E08.42, 250.21, E13.11, O24.425, O24.429, E11.3491, E11.3392, E08.3393, E11.620, E11.618, EHGZ5, E08.649, E13.618, E11.3591, E11.349, E11.3391, E11.3551, E11.339, O24.439, E13.52, E09.11, E09.22, E08.52, E09.621, E13.69, 250.6, 295, E08.341, E11.3319, E08.331, E10.3319, E08.3413, E08.3313, E11.3493, 249.10, 249.90, 250.23, E10.3493, E10.43, E11.331, E13.21, E08.36, E08.59, E08.43, E09.40, E08.329, E08.321, E09.59, E09.21, E11.3419, E13.59, 249.50, E10.622, E10.3312, E10.3311, 249.41, E10.321, E09.329, E08.3519, E08.618, E08.3593, E13.8, E09.69, E09.8, E09.42, E08.11, E10.3292, 250.53, E13.628, E10.3593, E10.628, E10.351, E10.3519, E10.3591, E13.00, E08.3513, E10.3511, E10.3399, E10.3513, E10.3599, E11.3312, E08.3299, E10.359, E08.49, E09.649, E09.43, E10.41,E13.43
Coronary artery disease	414.00, 411.81, 414.9, 411.89, 414.01, 414.8, 414.0, 440.21, 440.1, 414.05, 440.9, 440.20, I20.9, I25.10, I20.8, I25.9, I24.8, I25.110, I25.119, I20.0, I70.0, I25.118, I25.709, I70.739, MLAN9, LPA91, MLPG1, 198, 133, 132, I25.810, I70.211, I70.262, I70.233, I70.249, I70.25, I70.8, I70.219, I70.261, 414.2, I25.708, I70.245, I70.209, I70.1, I70.213, I70.212, 437.0, I70.90, I25.758, I25.759, LPA32, 440.8, 440.0, I70.434, I70.229, I25.83, I70.299, I70.202, I70.444, I70.221, I70.203, 414.04, I67.2, I24.9, I70.292, 440.29, 414.3, 440.23, I25.710, 440.22, I25.82, I25.720, I25.700, I70.242, 414.02, I25.84, I70.244, I70.448, I70.445, I70.409, I70.235, 414.06, 440.24, I25.799, I70.243, I25.811, I25.718, I25.89, MLEH1, MLPE4, I70.223, I70.232, I70.201, I70.569, I70.269, I70.629, I70.248, I25.111, I70.234, I70.208, I70.222, I70.302, I70.239, I70.263, I70.293, 440.30, I25.760, 414.03, I25.728, 440.2, I25.812, I25.798, MLAI5, NLHB1, I25.790, I20.1, I25.719, I70.91, 414, I25.701, 440.31, I70.411, I70.238, 440, I70.218, I70.313
Cirrhosis	571.5, 571.2, K70.30, K74.60, K74.69, QLJL1, 202, K70.31, K74.3, K74.5, LPA81, 571.6, K74.4, LPA1363, K71.7
Chronic obstructive pulmonary disease	J44.9, J44.0, J44.1, LPA80, LHAZ1, 088, 140, 492.8, J43.8, 494.1, J47.9, J47.1, J47.0, 494.0, LPA54, 494, 491.20, 491.22, 491.21, 748.61, LJFH8, 491.2
Congestive heart failure	544, 428.0, LPA89, MLSN3, 404.91, 402.91, 402.11, 402.00, 404.11, 404.13, 404.93, 404.01

Human immunodeficiency virus	B20, Z21, 042, V08, LPA190, 894, 489, 893, 892, 490, DJBQ4, 079.53, 795.71, 795.8, 890, DKNF1, 488, Z71.7, 042.0

Supplemental Table S3. Medication list

Medication class	Medication names
Angiotensin converting enzyme inhibitors / angiotensin II receptor blockers	benazepril, lotensin, captopril, capoten, "enalapril", "vasotech", "epaned", "lexxel", "fosinopril", "monopril", "lisinopril", "prinvil", "zestril", "qbrelis", "moexipril", "univasc", "perindopril", "aceon", "quinapril", "accupril", "ramipril", "altace", "trandolapril", "mavik", "azilsartan", "edarbi", "candesartan", "atacand", "eprosartan", "teveten", "irbesartan", "avapro", "telmisartan", "micardis", "valsartan", "diovan", "losartan", "cozaar", "olmesartan", "benicar", "lotrel", "hyzaar"
Loop diuretics	"Bumetanide", "Bumex", "Ethacrynic acid", "Edecrin", "Furosemide", "Lasix", "Torsemide", "Demadex"
Thiazide diuretics	"Hydrochlorothiazide","hctz","benicar","chlorathiazide","chlorthalidone","diuril","hyzaar","indapamide","metolazone","micardis","prinzide"
Potassium-sparing diuretics	"Aldactone","Amiloride","Dyazide","Eplerenone","Maxzide","Spironolactone","Triamterene"
Proton-pump inhibitors	"omeprazole", "Prilosec", "Yosprala", "lansoprazole", "Prevacid", "Prevacid 24-Hour", "dexlansoprazole", "Dexilent", "rabeprazole", "Aciphex", "pantoprazole", "Protonix", "esomeprazole", "Nexium", "Vimovo", "Zegerid", "dexilant", "dexlansoprazole"
Non-steroidal anti-inflammatory drugs	"celecoxib","celebrex","diclofenac","cambia","cataflam","voltaren","zipsor","zorvolex","diflunisal","dolobid","etodolac","lodine","ibuprofen","motrin","advil","idomethacin","indocin","ketoprofen","orudis","ketorolac","toradol","nabumetone","relafen","naproxen","aleve","anaprox","naprelan","naprosyn","oxaprozin","daypro","piroxicam","feldene","salsalate","disalsate","amigesic","sulindac","clinoril","tolmetin","tolectin"
Immunosuppressants	"jakafi", "ruxolitinib", "tofacitinib", "xeljanz", "cyclosporine", "neoral", "sandimmune", "sangcya", "tacrolimus", "astagraf", "envarsus", "prograf", "sirolimus", "rapamune", "everolimus", "afinitor", "zortress", "azathioprine", "azasan", "imuran", "leflunomide", "arava", "mycophenolate", "cellcept", "myfortic", "abatacept", "orencia", "adalimumab", "humira", "anakinra", "kineret", "certolizumab", "cimzia", "etanercept", "enbrel", "golimumab", "simponi", "infliximab", "remicade", "ixekizumab", "taltz", "natalizumab", "tysabri", "rituximab", "rituxan", "secukinumab", "cosentyx", "tocilizumab", "actemra", "ustekinumab", "stelara", "vedolizumab", "entyvio", "basiliximab", "simulect", "daclizumab", "zinbryta", "antithymocyte globulin", "IVIG", "methotrexate", "prednisone", "deltasone", "orasone", "budesonide", "entocort", "prograf", "astagraf", "mycophenolate", "rapamune", "afinitor", "imuran", "arava", "cyclophosphamide","eculizumab", "soliris", “belatacept”, “nulojix”

Supplemental Table S4. Missing laboratory values at hospital admission

Laboratory test	COVID-19 (n=251)	Influenza (n=179)
Creatinine	0	1 (0.6%)
White blood cell count	0	1 (0.6%)
Albumin	1 (0.4%)	24 (13.4%)
Hemoglobin	0	1 (0.6%)
Platelets	0	1 (0.6%)
Hematocrit	0	1 (0.6%)
Sodium	1 (0.4%)	1 (0.6%)
Potassium	1 (0.4%)	1 (0.6%)
Chloride	1 (0.4%)	1 (0.6%)
Bicarbonate	1 (0.4%)	1 (0.6%)
BUN	1 (0.4%)	1 (0.6%)
Neutrophils %	1 (0.4%)	2 (1.1%)
ALT	1 (0.4%)	26 (14.5%)
AST	1 (0.4%)	24 (13.4%)
Total bilirubin	1 (0.4%)	24 (13.4%)
Lactate	46 (18.3%)	39 (21.8%)
PT-INR	47 (18.7%)	63 (35.2%)
PTT	111 (44.2%)	132 (73.7%)

Supplemental Table S4. Abbreviations: ALT = alanine aminotransferase; AST = aspartate aminotransferase; BUN = blood urea nitrogen; PT-INR = prothrombin time and international normalized ratio; PTT = partial thromboplastin time (PTT); WBC = white blood cell count.

Supplemental Table S5. Standardized differences between baseline characteristics in non-propensity score matching, propensity score matching, and IPTW cohorts

	Standardized difference		
	Before Propensity Score	Propensity Score Matching	IPTW
Age	-0.2109	0.0309	-0.0051
Baseline eGFR	0.2363	-0.0179	0.0191
Sex, Male	0.0921	0.0184	-0.0014
Race / ethnicity	0.5637	0.0483	0.0722
Hypertension	-0.2117	0	-0.0174
Diabetes mellitus	0.1041	0.0291	0.0252
Coronary artery disease	-0.3679	-0.0174	-0.0168
Chronic obstructive pulmonary disease	-0.5445	-0.0405	-0.0627
Congestive heart failure	-0.279	0.0077	-0.0246
Cirrhosis	-0.0503	-0.0174	-0.0168
Human immunodeficiency virus	-0.0133	-0.0427	-0.0147
ACEi/ARB	-0.0765	0	-0.0095
Loop diuretics	-0.2043	-0.0086	-0.0224
Thiazide diuretics	0.0002	-0.015	0.0024
K-sparing diuretics	-0.1129	0.0052	-0.0124
Proton pump inhibitors	-0.2292	-0.008	-0.0186
NSAIDs	0.0457	-0.0028	-0.0138
Immunosuppressants	-0.5078	-0.0125	-0.0767
Hemoglobin	0.2394	0.0729	0
White blood cell count	0.2161	0.0509	0.0511
Sodium	0.1031	0.0953	0.0289
Albumin	0.2534	0.0314	0.0335
Platelets	0.2113	0.0782	0

Supplemental Table S5. Abbreviations: IPTW = inverse probability treatment weighting; eGFR = estimated glomerular filtration rate; ACEi/ARB = angiotensin converting enzyme inhibitors / angiotensin II receptor blockers; NSAID = non-steroidal anti-inflammatory drug.

Supplemental Table S6. Comparison of additional laboratory tests in patients with COVID-19 and influenza related acute kidney injury

Laboratory test, units	COVID-19 (n=251) median (IQR)	Influenza (n=179) median (IQR)	Missing data among 251 patients with COVID-19 AKI n (%)	Missing data among 179 patients with influenza AKI n (%)
D-dimer, µg/mL	1.19 (0.78 – 2.58)	0.96 (0.96 – 1.89)	168 (67%)	176 (98%)
Microalbumin-creatinine ratio, mg/g Cre	396 (98 – 999)	130.6 (130.6 – 130.6)	237 (94%)	178 (99%)
Total protein-creatinine ratio	0.38 (0.24 – 1.06)	0.25 (0.22 – 0.46)	229 (91%)	176 (98%)
C-reactive protein, mg/L	81 (42 – 147)	131 (43 – 149)	15 (6%)	163 (91%)
Arterial pCO2, mmHg	39 (33 – 45)	41 (33 – 49)	157 (63%)	143 (80%)
Venous pCO2, mmHg	41 (35 – 49)	46 (39 – 57)	106 (42%)	100 (56%)
IL-6, pg/mL	55.4 (25.4 – 118.5)	–	165 (66%)	179 (100%)
LDH, U/L	346 (270 – 487)	385 (246 – 487)	10 (4%)	145 (81%)

Supplemental Table S6. All variables included in this table have a large fraction of missing data. Abbreviations: IL-6 = interleukin-6; LDH = lactate dehydrogenase, CO2= carbon dioxide

Supplemental Table S7A. Acute kidney injury Stage 2 or higher among hospitalized patients (Multivariable Cox proportional hazards model)

Variable	aHR	95% CI	*P*-value
Age	0.99	0.98 – 1.00	0.16
Sex, male	1.15	0.85 – 1.55	0.35
Baseline creatinine	1.06	0.75 – 1.51	0.73
Race			
White	0.88	0.59 – 1.30	0.52
**Hispanic**	**0.58**	**0.35 – 0.96**	**0.03**
Black	-	-	-
Other	1.15	0.66 – 2.01	0.62
**COVID-19 vs influenza**	**2.09**	**1.50 – 2.91**	**<0.01**
Comorbidities			
Hypertension	1.48	0.89 – 2.47	0.13
**Diabetes mellitus**	**1.56**	**1.16 – 2.11**	**<0.01**
COPD	0.91	0.63 – 1.30	0.60
Medications			
**ACEi/ARBs**	**1.50**	**1.10 – 2.04**	**0.01**
Thiazide diuretics	1.23	0.85 – 1.77	0.27
**PPIs**	**0.66**	**0.49 – 0.90**	**<0.01**

Supplemental Table S7A. Abbreviations: COVID-19 = coronavirus disease 2019; COPD = chronic obstructive pulmonary disease; ACEi/ARB = angiotensin converting enzyme inhibitors / angiotensin II receptor blockers; PPI = proton pump inhibitor; aHR = adjusted hazard ratio; aHR = adjusted hazard ratio; CI = confidence interval.

Supplemental Table S7B. Acute kidney injury Stage 3 among hospitalized patients (Multivariable Cox proportional hazards model)

Variable	aHR	95% CI	*P*-value
Age	1.00	0.99 – 1.01	1.00
Sex, male	1.34	0.87 – 2.08	0.18
Baseline creatinine	0.94	0.56 – 1.58	0.82
Race			
White	0.62	0.36 – 1.06	0.08
**Hispanic**	**0.40**	**0.20 – 0.81**	**0.01**
Black	-	-	-
Other	0.94	0.44 – 2.02	0.88
**COVID-19 vs influenza**	**2.67**	**1.56 – 4.58**	**<0.01**
Comorbidities			
**Diabetes mellitus**	**1.98**	**1.27 – 3.10**	**<0.01**
COPD	0.68	0.37 – 1.24	0.20
**Cirrhosis**	**2.15**	**1.09 – 4.24**	**0.03**
CAD	0.72	0.46 – 1.14	0.16

Supplemental Table S7B. Abbreviations: COVID-19 = coronavirus disease 2019; COPD = chronic obstructive pulmonary disease; CAD = coronary artery disease; aHR = adjusted hazard ratio; CI = confidence interval.

Supplemental Table S7C. In-hospital mortality among total cohort (Multivariable Cox proportional hazards model)

Variable	aHR	95% CI	*P*-value
**Age**	**1.04**	**1.03 – 1.06**	**<0.01**
Sex, male	1.15	0.84 – 1.58	0.39
Baseline creatinine	1.28	0.90 – 1.82	0.16
Race			
White	1.27	0.80 – 2.04	0.31
Hispanic	0.78	0.41 – 1.46	0.43
Black	-	-	-
Other	0.87	0.40 – 1.91	0.73
**COVID-19 vs influenza**	**7.17**	**4.78 – 10.76**	**<0.01**
Comorbidities			
Hypertension	0.99	0.57 – 1.74	0.98
**Diabetes mellitus**	**1.41**	**1.02 – 1.94**	**0.04**
Cirrhosis	0.33	0.10 – 1.03	0.06
Medications			
**Loop diuretics**	**1.54**	**1.12 – 2.13**	**<0.01**
NSAIDs	0.82	0.57 – 1.18	0.29

Supplemental Table S7C. Abbreviations: COVID-19 = coronavirus disease 2019; NSAID = non-steroidal anti-inflammatory drug; aHR = adjusted hazard ratio; CI = confidence interval.

Supplemental Table S7D. In-hospital mortality among patients with acute kidney injury Stage 1 or higher (Multivariable Cox proportional hazards model)

Variable	aHR	95% CI	*P*-value
**Age**	**1.04**	**1.02 – 1.05**	**<0.01**
Sex, male	0.74	0.47 – 1.16	0.19
**Baseline creatinine**	**1.71**	**1.08 – 2.71**	**0.02**
Race			
White	1.59	0.90 – 2.83	0.11
Hispanic	0.84	0.38 – 1.84	0.66
Black	-	-	-
Other	0.97	0.40 – 2.39	0.95
**COVID-19 vs influenza**	**3.55**	**2.11 – 5.97**	**<0.01**
Comorbidities			
Cirrhosis	0.27	0.07 – 1.12	0.07

Supplemental Table S7D. Abbreviations: COVID-19 = coronavirus disease 2019; aHR = adjusted hazard ratio; CI = confidence interval; aHR = adjusted hazard ratio; CI = confidence interval.

Supplemental Table S7E. Acute kidney injury recovery to within 20% of pre-hospital baseline creatinine by hospital discharge (Multivariable Cox proportional hazards model)

Variable	aHR	95% CI	*P*-value
Age	1.00	0.99 – 1.01	0.86
Sex, male	1.09	0.84 – 1.42	0.51
**Baseline creatinine**	**0.62**	**0.46 – 0.83**	**<0.01**
Race			
White	0.95	0.67 – 1.34	0.76
**Hispanic**	**1.62**	**1.05 – 2.48**	**0.03**
Black	-	-	-
Other	0.96	0.55 – 1.68	0.88
**COVID-19 vs influenza**	**0.47**	**0.36 – 0.62**	**<0.01**
Comorbidities			
COPD	1.04	0.79 – 1.38	0.77
CAD	1.17	0.90 – 1.52	0.25

Supplemental Table S7E. Abbreviations: COVID-19 = coronavirus disease 2019; COPD = chronic obstructive pulmonary disease; CAD = coronary artery disease; aHR = adjusted hazard ratio; CI = confidence interval.

Supplemental Table S7F. Estimated glomerular filtration rate (eGFR) decline ≥ 25% at last follow-up among patients followed for ≥ 90 days (Multivariable Cox proportional hazards model)

Variable	aHR	95% CI	*P*-value
Age	1.00	0.99 – 1.01	0.91
Sex, male	1.01	0.73 – 1.39	0.97
Baseline creatinine	1.04	0.71 – 1.50	0.85
Race			
**White**	**0.63**	**0.41 – 0.97**	**0.04**
**Hispanic**	**0.27**	**0.13 – 0.53**	**<0.01**
Black	-	-	-
Other	0.94	0.50 – 1.77	0.85
COVID-19 vs influenza	1.36	0.97 – 1.90	0.07
Comorbidities			
Hypertension	1.40	0.73 – 2.66	0.31
Medications			
ACEi/ARBs	1.33	0.96 – 1.85	0.09
**Loop diuretics**	**1.74**	**1.26 – 2.41**	**<0.01**
**Immunosuppressants**	**0.65**	**0.46 – 0.92**	**0.02**

Supplemental Table S7F. Only patients surviving to hospital discharge who had their serum creatinine measured ≥ 90 days from diagnosis of COVID-19 or influenza were included in this analysis. Patients were followed for up to 10 months post-diagnosis. Median follow-up was 236 days (IQR 175 – 280) for patients with COVID-19 and 252 days (IQR 194 – 288) for patients with influenza included in this analysis. Abbreviations: COVID-19 = coronavirus disease 2019; ACEi/ARB = angiotensin converting enzyme inhibitors / angiotensin II receptor blockers; aHR = adjusted hazard ratio; CI = confidence interval.

Supplemental Table S7G. New-onset chronic kidney disease among patients followed for ≥ 90 days (Multivariable Cox proportional hazards model)

Variable	aHR	95% CI	*P*-value
**Age**	**1.04**	**1.03 – 1.06**	**<0.01**
**Sex, male**	**0.19**	**0.12 – 0.30**	**<0.01**
**Baseline creatinine**	**1.94**	**1.69 – 2.23**	**<0.01**
Race			
**White**	**3.21**	**1.80 – 5.72**	**<0.01**
Hispanic	1.66	0.76 – 3.60	0.20
Black	-	-	-
**Other**	**2.99**	**1.32 – 6.78**	**<0.01**
COVID-19 vs influenza	1.24	0.86 – 1.78	0.26
Comorbidities			
Hypertension	1.01	0.51 – 1.99	0.98
**Diabetes mellitus**	**1.65**	**1.16 – 2.33**	**<0.01**
COPD	1.07	0.74 – 1.55	0.71
CHF	1.18	0.75 – 1.85	0.48
**Cirrhosis**	**2.09**	**1.17 – 3.74**	**0.01**
**CAD**	**1.69**	**1.14 – 2.51**	**<0.01**
Medications			
ACEi/ARBs	1.23	0.86 – 1.74	0.26
**Loop diuretics**	**1.53**	**1.06 – 2.23**	**0.03**
K-sparing diuretics	1.24	0.72 – 2.13	0.43

Supplemental Table S7G. Only patients surviving to hospital discharge who had their serum creatinine measured ≥ 90 days from diagnosis of COVID-19 or influenza were included in this analysis. Patients were followed for up to 10 months post-diagnosis. Median follow-up was 232 days (IQR 176 – 280) for patients with COVID-19 and 252 days (IQR 193 – 287) for patients with influenza included in this analysis. Abbreviations: COVID-19 = coronavirus disease 2019; COPD = chronic obstructive pulmonary disease; CHF = congestive heart failure; CAD = coronary artery disease; ACEi/ARB = angiotensin converting enzyme inhibitors / angiotensin II receptor blockers; aHR = adjusted hazard ratio; CI = confidence interval.

Supplemental Table S8A. Acute kidney injury Stage 1 or higher among hospitalized patients (Multivariable Cox proportional hazards model, propensity score cohort)

Variable	aHR	95% CI	*P*-value
**Age**	**0.99**	**0.98 – 1.00**	**0.03**
Sex, male	1.05	0.82 – 1.36	0.68
Baseline creatinine	0.91	0.68 – 1.22	0.54
Race			
White	0.73	0.52 – 1.03	0.07
**Hispanic**	**0.58**	**0.35 – 0.96**	**0.04**
Black	-	-	-
Other	0.60	0.34 – 1.04	0.07
**COVID-19 vs influenza**	**1.70**	**1.32 – 2.20**	**<0.01**
Comorbidities			
**Hypertension**	**2.11**	**1.31 – 3.39**	**<0.01**
Diabetes mellitus	1.24	0.96 – 1.60	0.10
Cirrhosis	1.51	0.98 – 2.35	0.07
Medications			
**ACEi/ARBs**	**1.30**	**1.00 – 1.69**	**0.05**
**Loop diuretics**	**1.37**	**1.04 – 1.82**	**0.03**
Thiazide diuretics	1.05	0.76 – 1.45	0.79
K-sparing diuretics	1.27	0.84 – 1.91	0.26
PPIs	0.98	0.76 – 1.26	0.87

Supplemental Table S7A. This propensity score matching cohort included 1520 individuals (63% of total cohort), 760 from each cohort. Matching is described in the Methods section of the main manuscript. Abbreviations: COVID-19 = coronavirus disease 2019; ACEi/ARB = angiotensin converting enzyme inhibitors / angiotensin II receptor blockers; PPI = proton pump inhibitor; aHR = adjusted hazard ratio; CI = confidence interval.

Supplemental Table S8B. Acute kidney injury Stage 1 or higher among hospitalized patients (Multivariable Cox proportional hazards model, propensity score inverse probability treatment weighting cohort)

Variable	aHR	95% CI	*P*-value
**Age**	**0.99**	**0.99 – 1.00**	**0.02**
Sex, male	1.07	0.87 – 1.31	0.52
Baseline creatinine	0.92	0.72 – 1.17	0.47
Race			
White	0.81	0.62 – 1.07	0.14
**Hispanic**	**0.62**	**0.42 – 0.90**	**0.01**
Black	-	-	-
Other	0.75	0.49 – 1.16	0.20
**COVID-19 vs influenza**	**1.67**	**1.38 – 2.04**	**<0.01**
Comorbidities			
**Hypertension**	**1.95**	**1.31 – 2.90**	**<0.01**
**Diabetes mellitus**	**1.25**	**1.02 – 1.53**	**0.03**
**Cirrhosis**	**1.43**	**1.00 – 2.05**	**0.05**
Medications			
**ACEi/ARBs**	**1.53**	**1.24 – 1.88**	**<0.01**
**Loop diuretics**	**1.35**	**1.08 – 1.68**	**0.01**
Thiazide diuretics	1.01	0.78 – 1.30	0.93
**K-sparing diuretics**	**1.46**	**1.08 – 1.97**	**0.02**
PPI	0.98	0.80 – 1.20	0.85

Supplemental Table S8B. This propensity score matching with inverse probability treatment weighting (IPTW) cohort included 2400 individuals (99% of original cohort). Abbreviations: COVID-19 = coronavirus disease 2019; ACEi/ARB = angiotensin converting enzyme inhibitors / angiotensin II receptor blockers; PPI = proton pump inhibitor; aHR = adjusted hazard ratio; CI = confidence interval.

STROBE Statement—Checklist of items that should be included in reports of ***cohort studies***

	Item No	Recommendation	Page No
**Title and abstract**	1	(*a*) Indicate the study's design with a commonly used term in the title or the abstract<br>(*b*) Provide in the abstract an informative and balanced summary of what was done and what was found	1-2
**Introduction**			
Background/rationale	2	Explain the scientific background and rationale for the investigation being reported	3
Objectives	3	State specific objectives, including any prespecified hypotheses	3
**Methods**			
Study design	4	Present key elements of study design early in the paper	3
Setting	5	Describe the setting, locations, and relevant dates, including periods of recruitment, exposure, follow-up, and data collection	3
Participants	6	(*a*) Give the eligibility criteria, and the sources and methods of selection of participants. Describe methods of follow-up<br>(*b*) For matched studies, give matching criteria and number of exposed and unexposed	3-4
Variables	7	Clearly define all outcomes, exposures, predictors, potential confounders, and effect modifiers. Give diagnostic criteria, if applicable	4
Data sources/ measurement	8*	For each variable of interest, give sources of data and details of methods of assessment (measurement). Describe comparability of assessment methods if there is more than one group	3
Bias	9	Describe any efforts to address potential sources of bias	3
Study size	10	Explain how the study size was arrived at	4
Quantitative variables	11	Explain how quantitative variables were handled in the analyses. If applicable, describe which groupings were chosen and why	4
Statistical methods	12	(*a*) Describe all statistical methods, including those used to control for confounding<br>(*b*) Describe any methods used to examine subgroups and interactions<br>(*c*) Explain how missing data were addressed<br>(*d*) If applicable, explain how loss to follow-up was addressed<br>(*e*) Describe any sensitivity analyses	4-5
**Results**			
Participants	13*	(a) Report numbers of individuals at each stage of study—eg numbers potentially eligible, examined for eligibility, confirmed eligible, included in the study, completing follow-up, and analysed<br>(b) Give reasons for non-participation at each stage<br>(c) Consider use of a flow diagram	5
Descriptive data	14*	(a) Give characteristics of study participants (eg demographic, clinical, social) and information on exposures and potential confounders<br>(b) Indicate number of participants with missing data for each variable of interest<br>(c) Summarise follow-up time (eg, average and total amount)	5
Outcome data	15*	Report numbers of outcome events or summary measures over time	6-7

Main results	16	(*a*) Give unadjusted estimates and, if applicable, confounder-adjusted estimates and their precision (eg, 95% confidence interval). Make clear which confounders were adjusted for and why they were included<br>(*b*) Report category boundaries when continuous variables were categorized<br>(*c*) If relevant, consider translating estimates of relative risk into absolute risk for a meaningful time period	6-7
Other analyses	17	Report other analyses done—eg analyses of subgroups and interactions, and sensitivity analyses	6-7
**Discussion**			
Key results	18	Summarise key results with reference to study objectives	7
Limitations	19	Discuss limitations of the study, taking into account sources of potential bias or imprecision. Discuss both direction and magnitude of any potential bias	8-9
Interpretation	20	Give a cautious overall interpretation of results considering objectives, limitations, multiplicity of analyses, results from similar studies, and other relevant evidence	9
Generalisability	21	Discuss the generalisability (external validity) of the study results	9
**Other information**			
Funding	22	Give the source of funding and the role of the funders for the present study and, if applicable, for the original study on which the present article is based	9

*Give information separately for exposed and unexposed groups.

Note: An Explanation and Elaboration article discusses each checklist item and gives methodological background and published examples of transparent reporting. The STROBE checklist is best used in conjunction with this article (freely available on the Web sites of PLoS Medicine at http://www.plosmedicine.org/, Annals of Internal Medicine at http://www.annals.org/, and Epidemiology at http://www.epidem.com/). Information on the STROBE Initiative is available at http://www.strobe-statement.org.
